# Stochastic Modeling and Analysis of Multiple Nonlinear Accelerated Degradation Processes through Information Fusion

**DOI:** 10.3390/s16081242

**Published:** 2016-08-06

**Authors:** Fuqiang Sun, Le Liu, Xiaoyang Li, Haitao Liao

**Affiliations:** 1 Science and Technology on Reliability and Environmental Engineering Laboratory, School of Reliability and Systems Engineering, Beihang University, Beijing 100191, China; sunfuqiang@buaa.edu.cn (F.S.); liule@buaa.edu.cn (L.L.); leexy@buaa.edu.cn (X.L.); 2 Department of Industrial Engineering, University of Arkansas, Fayetteville, AR 72701, USA

**Keywords:** accelerated degradation testing, nonlinearity, general Wiener process, multiple performance parameters, copulas, *s*-dependency

## Abstract

Accelerated degradation testing (ADT) is an efficient technique for evaluating the lifetime of a highly reliable product whose underlying failure process may be traced by the degradation of the product’s performance parameters with time. However, most research on ADT mainly focuses on a single performance parameter. In reality, the performance of a modern product is usually characterized by multiple parameters, and the degradation paths are usually nonlinear. To address such problems, this paper develops a new *s*-dependent nonlinear ADT model for products with multiple performance parameters using a general Wiener process and copulas. The general Wiener process models the nonlinear ADT data, and the dependency among different degradation measures is analyzed using the copula method. An engineering case study on a tuner’s ADT data is conducted to demonstrate the effectiveness of the proposed method. The results illustrate that the proposed method is quite effective in estimating the lifetime of a product with *s*-dependent performance parameters.

## 1. Introduction

Modern products are developed to have good quality and high reliability. For some safety-critical components and systems, they are even designed to last for an extremely long time to avoid the catastrophic consequences of potential failures. It is a big challenge to obtain sufficient amount of time-to-failure data by testing such products under the normal operating environments and sometimes even under harsher conditions [1]. Obviously, the traditional failure-time data analysis and testing methods are unsuitable for reliability analysis of such highly reliable products.

In engineering applications, many failure mechanisms can be traced to underlying degradation processes (e.g., cumulative wear, crack growth, corrosion, fatigue, material aging, etc.) [2]. As a result, the degradation analysis method has been introduced to handle reliability modeling problems based on the products’ degradation information obtained from historical data or degradation tests [3]. Especially, accelerated degradation testing (ADT) has been used to collect the degradation data by exposing the test specimens to severer-than-normal conditions. In the last two decades, ADT has been intensively studied as an effective tool for reliability verification and lifetime evaluation of modern products. Successful applications of ADT include the reliability analyses of batteries [4], super luminescent diode (SLD) [5], smart electricity meter [6], metal oxide semiconductor field effect transistors (MOSFETs) [7], etc.

The essence of ADT-based decision-making is to find a suitable mathematical model, namely degradation model, which is capable of describing the degradation paths of samples tested at different stress levels. The existing degradation models can be categorized into two broad classes, i.e., stochastic process models and general path models [1]. The stochastic process models have attracted more attention because of their properties of time-dependent structures. The typical models include the Wiener process (i.e., Brownian motion), Gamma process and Inverse Gaussian (IG) process. The Gamma and IG processes are suitable for modeling a degradation process which is always positive and strictly increasing. On the other hand, the Wiener process with a linear drift has become the most popular stochastic degradation model [1,8]. Its related work and variants for the cases involving covariates, random effects and measurement errors have been extensively reviewed by Ye and Xie [1]. Nonetheless, the linear drift Wiener process cannot be directly used to describe a nonlinear degradation process. In many engineering applications, however, nonlinearity is quite natural due to the complex structures and failure mechanisms of the products. To overcome this obstacle, some transformation methods for degradation data have been used, e.g., time-scale transformation [9,10,11] and log-transformation [12,13,14]. However, not all nonlinear degradation processes can be properly transformed [15]. To characterize the dynamics and nonlinearity of the degradation process, Si et al. [15] developed a Wiener process model with a nonlinear drift coefficient, and presented analytical approximations to the probability distribution functions of the first hitting time under a mild assumption. Further, Wang et al. [16,17] presented a general Wiener process model for residual life estimation which jointly takes into account the nonlinearity, temporal uncertainty, and unit-to-unit variability. In this paper, we will first introduce the general Wiener process and then extend this model for analyzing nonlinear ADT data.

It is also worth pointing out that the aforementioned studies only consider the cases involving a single degradation measure. Indeed, modern engineering systems are often composed of multiple components with different functions [18]. As a result, a product may have multiple degradation measures and any of them may be a cause of product failure [19]. Such degradation measures may include not only the functional and/or performance parameters of the product, but also some indirect degradation features extracted from raw sensory signals [20], such as vibration, force and acoustic signals, temperature and voltage. Usually, degradation measures are not independent of each other, and some failures may be attributed to the interaction of multiple degradation processes. In practice, it is often difficult to determine the importance of each source of degradation information. To avoid possible one-sidedness, it would be more appropriate and accurate to perform reliability estimation through suitable fusion of information from different degradation measures. When modeling ADT data, such efforts become more challenging as the data are collected under different stress levels.

In the last decade, some progress has been made on multivariate degradation modeling. Huang and Askin [21] discussed competing modes of catastrophic failure and degradation failure by assuming the independence of multiple degradation processes. Crowder [22] suggested that such an independence assumption can only be made if a failure mechanism has absolutely no direct or indirect impact on the likelihood of other failure mechanisms in the system. He also pointed out that some shared factors (e.g., same environmental/operational stresses, usage history, materials quality, and maintenance of the system) may increase the possibility of dependence of different failure mechanisms in the system. Therefore, it is safer to consider the dependency between performance parameters in a multivariate degradation model.

In the literature, two popular methods have been utilized to capture the dependence between performance parameters, namely, the use of multivariate joint distributions [23,24,25] and the copulas method [18,19,26,27,28,29,30,31,32,33]. In practice, assuming a multivariate joint distribution may not be suitable for all conditions [34], and sometimes it is difficult, if not impossible, to find an appropriate joint distribution in many cases. As a useful alternative, the use copulas enables estimating a multivariate joint distribution by combining the marginal distributions using a joint dependence structure [35]. Moreover, copulas do not impose constraints on the univariate marginal distributions [36]. Due to these advantages, the copulas method has attracted much attention in reliability engineering. Sari et al. [26] introduced a copula function to describe the correlation between two performance parameters, and combined it with a generalized linear model for bivariate constant-stress degradation data. Pan et al. [27,28] discussed the bivariate degradation modeling approaches based on Wiener processes and Copulas, respectively, under the normal operating stress and constant-stress accelerating conditions as well. Similarly, Peng et al. [18], Liu et al. [29] and Hao et al. [30,31] also adopted copulas to characterize the dependency between two performance parameters, and developed their own bivariate degradation models. Wang and Pham [19] applied time-varying copulas for handling the *s*-dependent relationship among competing degradation processes, and provided a numerical example with two degradation processes. Hong et al. [32] used copulas to investigate the influence of degradation dependency of multiple components on the optimal maintenance decisions. Xi et al. [33] developed a copula-based sampling method for data-driven prognostics. However, most of the previous work focuses on degradation processes under normal operating conditions, and scarce studies have been conducted on the dependency of multiple degradation processes under accelerated conditions.

This paper is aimed at making an early attempt to model *s*-dependent multivariate ADT data using general Wiener process and copulas. First, the general Wiener process is used to model nonlinear univariate accelerated degradation processes. Then, the copula method is adopted to find the joint probability distribution of multiple degradation processes. Next, parameter estimation of the proposed model is performed using the Inference Functions for Margins (IFM) method, and the Akaike Information Criterion (AIC) is employed to compare the goodness-of-fit of candidate models. Finally, a real-world case study on the tuner’s CSADT data is utilized to demonstrate the usefulness of the proposed method.

The remainder of this paper is organized as follows: Section 2 presents the univariate ADT model based on the general Wiener process and its parameter estimation method. Section 3 elaborates on the copula-based multivariate ADT modeling method, including introduction to copulas, multivariate dependent accelerated degradation model, and statistical inference. Section 4 provides the case study, and Section 5 concludes the paper.

## 2. General Univariate Accelerated Degradation Model

As mentioned earlier, ADT attempts to obtain a product’s degradation information more quickly by exposing the test units to harsher-than-normal conditions. In many applications, two ADT methods have been widely used: constant-stress ADT (CSADT) and step-stress ADT (SSADT). For illustration purposes, this paper will focus on CSADT.

ADT modeling is concerned with accelerated degradation models and data analyses [37]. Generally, accelerated degradation model is composed of both stress-acceleration model and degradation model, where the stress-acceleration model describes the relationship between the levels of accelerating stress and the degradation rate, and the degradation model depicts the evolution of degradation process over time. In this section, a nonlinear univariate CSADT model based on the general Wiener process is presented.

### 2.1. Stress-Acceleration Model

To estimate the lifetime or degradation rate of a product at the use conditions, a stress-acceleration model should be obtained first. Usually, stress-acceleration model can be obtained from either some physical knowledge about the product or empirical observations. Some widely used models include Arrhenius model, Eyring model, and inverse power model [38]. After a proper transformation of stress *S_i_*, a stress-acceleration model for degradation rate *dr*(*S_i_*) of the product under stress level *S_i_* can be expressed as [11]:

*dr*(*S_i_*) = exp(*a* + *bϕ*(*S_i_*)) (1)

where *a* and *b* are unknown constants, and *ϕ*(*S_i_*) is a function of *S_i_* depending on the stress type, e.g., *ϕ*(*S_i_*) = 1/*S_i_* for absolute temperature and *ϕ*(*S_i_*) = ln*S_i_* for voltage.

### 2.2. Accelerated Degradation Model

#### 2.2.1. An ADT Model Based on the General Wiener Process

The general Wiener process used for degradation modeling can be expressed as [16]:

(2) $$X\left(t\right)=\mu \int_{0}^{t}\lambda \left(t;\theta \right)dt+\sigma B\left(\tau \left(t;\gamma \right)\right)+X(0)$$

where *X(t)* is the degradation value at time *t*; *X*(0) is the initial degradation value (for simplicity, we assume *X*(0) = 0); *B*(.) is the standard Brownian motion; *μ* is the drift coefficient reflecting the rate of degradation; *σ* is the diffusion parameter describing the unit-to-unit variability and variability due to operational and environmental conditions; *λ*(*t*; *θ*) and *τ*(*t*; *γ*) are the non-decreasing time-scale transformation which can describe the nonlinear property of the degradation process, and *θ* and *γ* are the corresponding parameters.

By further assuming that $\Lambda \left(t;\theta \right)=\int_{0}^{t}\lambda \left(t;\theta \right)dt$, we have:

*M*_0_: *X*(*t*) = *μ*Λ(*t*; *θ*) + *σB*(*τ*(*t*; *γ*)) (3)

Clearly, the basic Wiener process model [1], as shown in Equation (4), is a special case of Equation (3) when Λ(*t*; *θ*) = *τ*(*t*; *γ*) = Λ(*t*):

*M*_1_: *X*(*t*) = *μ*Λ(*t*) + *σB*(Λ(*t*)) (4)

For clarity, Equation (3) is called model *M*_0_, while Equation (4) is called model *M*_1_ in this paper.

In model *M*_0_, since *E*[*X*(*t*)] = *μ*Λ(*t*; *θ*) and *Var*[*X*(*t*)] = *σ*^2^*τ*(*t*; *γ*), one can see that *θ* reflects the behavior of the expectation of degradation pattern while *γ* reflects the behavior of the variance of degradation. In model *M*_1_, the two parameters are assumed to be the same.

When using model *M_0_* for ADT analysis, the drift coefficient *μ* is modeled as a function of stresses, i.e., as a stress-acceleration model [1,5,10]. According to Equation (1), we have:

*μ* = exp(*a* + *bϕ*(*S_i_*)) (5)

The corresponding lifetime distribution can be obtained based on the time when the degradation path *X(t)* exceeds the failure threshold *ω* for the first time, i.e., First Passage Time (FPT). According to the property of linear Wiener process, the probability density function (PDF) of FPT follows an inverse Gaussian distribution [39]:

(6) $$f\left(t\right)=\frac{\omega }{t\sqrt{2\pi \sigma^{2}t}}\exp\left(-\frac{{\left(\omega -\mu t\right)}^{2}}{2\sigma^{2}t}\right)$$

Hence, for model *M*_1_ considering the time-scale transformation, the PDF of FPT and the reliability function of the product can be easily obtained as:

(7) $$f_{1}\left(t\right)=\frac{\omega }{\Lambda \left(t\right)\sqrt{2\pi \sigma^{2}\Lambda \left(t\right)}}\exp\left(-\frac{{\left(\omega -\mu \Lambda \left(t\right)\right)}^{2}}{2\sigma^{2}\Lambda \left(t\right)}\right)\frac{d\Lambda \left(t\right)}{dt}$$

(8) $$R_{1}\left(t\right)=\Phi \left(\frac{\omega -\mu \Lambda \left(t\right)}{\sigma \sqrt{\Lambda \left(t\right)}}\right)-\exp\left(\frac{2\mu \omega }{\sigma^{2}}\right)\Phi \left(-\frac{\omega +\mu \Lambda \left(t\right)}{\sigma \sqrt{\Lambda \left(t\right)}}\right)$$

respectively. However, the analytical formula of PDF of FPT for model *M_0_*, where Λ(*t*; *θ*)≠*τ*(*t*; *γ*), cannot be directly obtained from the above equations.

#### 2.2.2. Derivation of Failure Time Distribution for Model *M_0_*

Let the time-scale transformation be *s* = *τ*(*t*; *γ*), so that *t* = *τ*^−1^(*s*; *γ*). We also define Λ(*t*; *θ*) =Λ (*τ*^−1^(*s*; *γ*), *θ*) = *ρ*(*s*; *θ*). As a result, Equation (3) becomes [16]:

*X*(*s*) = *μρ*(*s*; *θ*) + *σB*(*s*) (9)

We define:

(10) $$\kappa \left(s;\theta \right)=\mu \frac{d\rho \left(s;\theta \right)}{ds}$$

Under some mild assumptions, the PDF of stochastic process *X*(*s*) can be approximated by (according to Theorem 2 in Si et al. [15] p. 56):

(11) $$p_{0}\left(s\right)≅\frac{1}{\sqrt{2\pi s}}\left(\frac{\omega -\mu \rho \left(s;\theta \right)}{\sigma s}+\frac{\kappa \left(s;\theta \right)}{\sigma }\right)\cdot \exp\left(-\frac{{\left(\omega -\mu \rho \left(s;\theta \right)\right)}^{2}}{2\sigma^{2}s}\right)$$

Hence, the PDF of failure time when *X*(*t*) exceeds *ω* can be obtained by substituting *s* with *τ*(*t*; *γ*) as:

(12) $$p_{0}\left(t\right)≅\frac{1}{\sqrt{2\pi \tau \left(t;\gamma \right)}}\left(\frac{\omega -\mu \Lambda \left(t;\theta \right)}{\sigma \tau \left(t;\gamma \right)}+\frac{\kappa \left(\tau \left(t;\gamma \right);\theta \right)}{\sigma }\right)\cdot \exp\left(-\frac{{\left(\omega -\mu \Lambda \left(t;\theta \right)\right)}^{2}}{2\sigma^{2}\tau \left(t;\gamma \right)}\right)\frac{d\tau \left(t;\gamma \right)}{dt}$$

Equation (12) can be further approximated as:

(13) $$f_{0}\left(t\right)≅p_{0}\left(t\right)/\int_{0}^{+\infty }p_{0}\left(t\right)dt$$

and the corresponding Cumulative Distribution Function (CDF) of FPT is:

(14) $$F_{0}\left(t\right)≅\int_{0}^{t}f_{0}\left(t\right)dt$$

Suppose that Λ(*t*; *θ*) = *t^θ^* and *τ*(*t*; *γ*) = *t^γ^* , then Equation (12) becomes:

(15) $$p_{0}\left(t\right)=\frac{\gamma t^{\gamma -1}}{\sqrt{2\pi t^{\gamma }}}\left(\frac{\omega -\mu t^{\theta }}{\sigma t^{\gamma }}+\frac{\theta \mu t^{\theta -\gamma }}{\gamma \sigma }\right)\exp\left(-\frac{{\left(\omega -\mu t^{\theta }\right)}^{2}}{2\sigma^{2}t^{\gamma }}\right)=\frac{\left(\omega \gamma -\left(\gamma -\theta \right)\mu t^{\theta }\right)}{t\sqrt{2\pi \sigma^{2}t^{\gamma }}}\exp\left(-\frac{{\left(\omega -\mu t^{\theta }\right)}^{2}}{2\sigma^{2}t^{\gamma }}\right)$$

When *θ* = *γ* = 1, Equation (13) is an inverse Gaussian distribution as in Equation (6) since $\int_{0}^{+\infty }p_{0}\left(t\right)dt=1$. When Λ(*t*; *θ*) = *τ*(*t*; *γ*), Equation (13) is an inverse Gaussian distribution with time-scale transformation as in Equation (7). Thus, Equation (13) is the generalized PDF of FPT which can cover existing linear and time-scale transformation Wiener process model as its limiting cases.

Then, according to Equation (14), the reliability function that relies on *S*_0_ can be expressed as:

*R*_0_(*t*; *S*_0_) = 1 − *F*_0_(*t*; *S*_0_) (16)

### 2.3. Parameter Estimation of General ADT Model

In this section, we briefly provide a two-stage Maximum Likelihood Estimation (MLE) for unknown parameters in CSADT with a single performance parameter. The unknown parameters are in vector Θ = {*θ*, *γ*, *σ*, *a*, *b*}.

Suppose that a univariate CSADT with *K* stress levels has been conducted, S*_i_*, *i* = 1, …, *K* is indicated the *i*th accelerated stress level, and S_0_ is normal operating condition. There are *n_i_* specimens under stress level S*_i_*. Then, *X_ijk_* is the *k*th degradation value of unit *j* at the stress level *i* and *t_ijk_* is the corresponding measurement time, *i* = 1, 2, …, *K*; *j* = 1, 2, …, *n_i_*; *k* = 1, 2, …, *m_ij_*. Let $X_{ij}=(X_{ij1},X_{ij2},\ldots ,X_{ijm_{ij}})\prime$ and $t_{ij}=(\Lambda (t_{ij1};\theta ),\Lambda (t_{ij2};\theta ),\ldots ,\Lambda (t_{ijm_{ij}};\theta ))\prime$. According to the properties of Wiener process, **X***_ij_* follows a multivariate normal distribution:

(17) $$X_{ij}\sim N\left(\mu_{ij}t_{ij},\sigma^{2}Q_{ij}\right)$$

where:

(18) $$Q_{ij}=\left[\begin{array}{cccc} \tau \left(t_{ij1};\gamma \right) & \tau \left(t_{ij1};\gamma \right) & \cdots & \tau \left(t_{ij1};\gamma \right)\\ \tau \left(t_{ij1};\gamma \right) & \tau \left(t_{ij2};\gamma \right) & \cdots & \tau \left(t_{ij2};\gamma \right)\\ \vdots & \vdots & \ddots & \vdots \\ \tau \left(t_{ij1};\gamma \right) & \tau \left(t_{ij2};\gamma \right) & \cdots & \tau \left(t_{ijm_{ij}};\gamma \right) \end{array}\right]$$

Let $\mu =(\mu_{11},\ldots ,\mu_{1n_{1}},\ldots ,\mu_{K1},\ldots ,\mu_{Kn_{K}})$. The likelihood function of CSADT data can be easily obtained, and the corresponding log-likelihood function is:

(19) $$l\left(\mu ,\sigma ,\theta ,\gamma |X\right)=-\frac{\ln\left(2\pi \right)}{2}\sum_{i=1}^{K}\sum_{j=1}^{n_{i}}m_{ij}-\frac{1}{2}\sum_{i=1}^{K}\sum_{j=1}^{n_{i}}\ln\left|\sigma^{2}Q_{ij}\right|-\frac{1}{2}\sum_{i=1}^{K}\sum_{j=1}^{n_{i}}{\left(X_{ij}-\mu_{ij}t_{ij}\right)}^{\prime }\sigma^{-2}Q_{ij}^{-1}\left(X_{ij}-\mu_{ij}t_{ij}\right)$$

Taking the first partial derivatives of Equation (19) with respect to *μ_ij_* and *σ*^2^ and setting the resulting equations to zero, the estimates ${\hat{\mu }}_{ij}$ and ${\hat{\sigma }}^{2}$ relying on *θ* and *γ* can be obtained as:

(20) $${\hat{\mu }}_{ij}=\frac{{X^{\prime }}_{ij}Q_{ij}^{-1}t_{ij}}{{t^{\prime }}_{ij}Q_{ij}^{-1}t_{ij}},\;{\hat{\sigma }}^{2}=\frac{\sum_{i=1}^{K}\sum_{j=1}^{n_{i}}{\left(X_{ij}-{\hat{\mu }}_{ij}t_{ij}\right)}^{\prime }Q_{ij}^{-1}\left(X_{ij}-{\hat{\mu }}_{ij}t_{ij}\right)}{\sum_{i=1}^{K}\sum_{j=1}^{n_{i}}m_{ij}}$$

Substituting Equation (20) into Equation (19), the log-likelihood function is only a function of *θ* and *γ*:

(21) $$l\left(\theta ,\gamma |X\right)=-\frac{\ln\left(2\pi \right)}{2}\sum_{i=1}^{K}\sum_{j=1}^{n_{i}}m_{ij}-\frac{1}{2}\sum_{i=1}^{K}\sum_{j=1}^{n_{i}}\ln\left|{\hat{\sigma }}^{2}Q_{ij}\right|-\frac{1}{2}\sum_{i=1}^{K}\sum_{j=1}^{n_{i}}{\left(X_{ij}-{\hat{\mu }}_{ij}t_{ij}\right)}^{\prime }{\hat{\sigma }}^{-2}Q_{ij}^{-1}\left(X_{ij}-{\hat{\mu }}_{ij}t_{ij}\right)$$

The estimates $\hat{\theta }$ and $\hat{\gamma }$ can be obtained using the two-dimensional search [40] that maximizes Equation (21). Then, ${\hat{\mu }}_{ij}$ and ${\hat{\sigma }}^{2}$ can be easily computed from Equation (20).

Finally, given the ${\hat{\mu }}_{ij}$s and the corresponding stress *S_i_*, the values of $\hat{a}$ and $\hat{b}$ can be easily computed from Equation (5) by the regression analysis.

## 3. General ADT Modeling through Multivariate Information Fusion

### 3.1. Copulas

**Figure 1:**
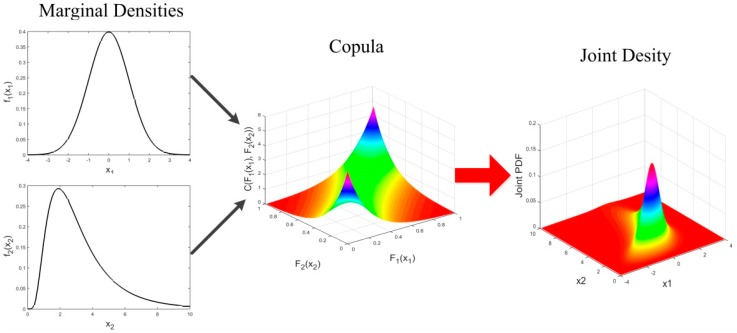
The multivariate dependence structure represented by a copula.

Identifying a multivariate probability distribution is difficult due to the complexity of marginal distributions and the curse of dimensionality. Copulas simplify this process by separating the learning of the marginal distributions from the learning of the multivariate dependence structure that links the marginal distributions to form a joint distribution [35], as shown in Figure 1.

The following theorem provides the necessary and sufficient condition for the existence of Copula, which elucidates the role that copulas play in describing the relationship between a multivariate distribution and the associated univariate marginal distributions.

**Theorem 1.**  (*Sklar’s theorem* [41]): Let *X* = (*X*_1_, …, *X_N_*) be a random vector with marginal distributions *F*_1_(*x*_1_), …, *F_N_*(*x_N_*), and let *F* be their joint distribution function. Then, there exists a copula function *C* such that:

(22) $$F\left(x_{1},\ldots ,x_{N}\right)=C\left(F_{1}\left(x_{1}\right),\ldots ,F_{N}\left(x_{N}\right)\right)$$

If *F_i_*(*x_i_*), *i* = 1, …, *N*, are continuous, the copula *C* is unique. Conversely, if *F*_1_(*x*_1_), …, *F_N_*(*x_N_*) are univariate distributions, the function *F* defined by Equation (22) is the joint distribution function of margins *F*_1_(*x*_1_), …, *F_N_*(*x_N_*).

According to the theorem, a multivariate Copula function can be defined as follows:

**Definition 1.**  *(N-dimensional copula, or N-copula)* [36]: An *N*-dimensional copula is a function *C* from **I***^N^* = [0,1]*^N^* to **I** with the following properties:

(1) Grounded: for every **u** = (*u*_1_, …, *u_N_*) in **I***^N^*, *C*(**u**) = 0 if at least one coordinate of **u** is 0;
(2) Uniform margins: if all coordinates of **u** are 1 except *u_k_*, then:
  *C*(**u**) = *C*(1,…,1, *u_k_*, 1,…,1) = *u_k_* (23)
(3) *N*-increasing: for each hyperrectangle $B=\overset{N}{\underset{i=1}{\Pi }}\left[u_{i},v_{i}\right]\subseteq {\left[0,1\right]}^{N}$, the *C*-volume of *B* is non-negative:
  (24) $$\int_{B}dC([u,v])=\sum_{z\in \times_{i=1}^{N}\{u_{i},v_{i}\}}{\left(-1\right)}^{N\left(z\right)}C(z)\geq 0$$
  where *N*(**z**) = #{*k*: *z_k_* = *u_k_*}.

The density of a copula function *C* is denoted by *c*, which may be achieved by taking partial derivative as:

(25) $$c\left(u_{1},\ldots ,u_{N}\right)=\frac{\partial^{N}C\left(u_{1},\ldots ,u_{N}\right)}{\partial u_{1}\ldots \partial u_{N}}\;\forall u=\left(u_{1},\ldots ,u_{N}\right)\in I^{N}$$

Furthermore, the joint density function corresponding to distribution function *F*(*x*_1_, …, *x_N_*) can be calculated by:

(26) $$f\left(x_{1},\ldots ,x_{N}\right)=c\left(F_{1}\left(x_{1}\right),\ldots ,F_{N}\left(x_{N}\right)\right)\prod_{n=1}^{N}f_{n}\left(x_{n}\right)$$

where *f_n_*(·), *n* = 1, …, *N*, is the PDF of marginal distribution *F_n_*(·).

Copulas have several attractive properties [42]. First, copulas provide a convenient way to model the marginal distributions and the joint dependence structure separately. Second, they are invariant under increasing and continuous transformations. Third, they have no constraints on the univariate marginal distribution. Because of these advantages, copulas are chosen to emphasize and measure the dependence among multiple degradation processes in this paper.

Table 1 lists several commonly used multivariate copula functions, where *u*_1_, …, *u_N_* denote univariate marginal distributions. The Akaike Information Criterion (AIC) will be employed to implement goodness-of-fit test and quantitatively select the most suitable copula type from several candidate copulas. In particular, the AIC is defined as:

*AIC* = 2*k* − 2ln*L* (27)

where *k* is the number of estimated parameters in the model, and *L* is the maximum likelihood value for the model. Given a set of candidate copulas for the multivariate accelerated degradation processes, the preferred copula function is the one with the minimum AIC value.

### 3.2. Multivariate Dependent Accelerated Degradation Model

In this section, a multivariate accelerated degradation model is presented to estimate the system reliability under the normal operating conditions. First, the CSADT data of each performance characteristic is modeled by a general Wiener process (see Section 2.2), and general accelerated degradation models for multiple performance parameters are established respectively. Then, copulas are utilized to describe the dependence among multiple performance parameters and conduct multi-parameters degradation information fusion.

To analyze the multivariate CSADT data, the following assumptions are made:

*A1*. The degradation measurements of all specimens are taken at the same time.
*A2*. A specimen is considered to be failed if one of the features reaches its corresponding failure threshold for the first time.
*A3*. The degradation processes of all performance parameters in CSADT can be depicted by the general Wiener process.
*A4*. For a dependent system with multiple performance parameters, the dependency among the multiple performance parameters can be characterized by copulas.

Suppose that a system has *N* degradation measures. Let *X_p_*(*t*), *p* = 1, 2, …, *N* denote the degradation process of the *p*th performance parameter at normal operating condition *S*_0_. According to above assumptions, the system is failed if any one performance parameter reaches its corresponding failure threshold represented by *D_p_*. Let the failure time of the *p*th characteristic index be *T_p_*, then the system lifetime is *T* = min(*T*_1_, …, *T_N_*). So, the system reliability under *S*_0_ can be expressed as:

*R*(*t*) = *P*(*T* > *t*) = *P*(*T*_1_ > *t*,…,*T_N_* > *t*) = *P*(*X*_1_ (*t*) < *D*_1_,…, *X_N_* (*t*) < *D_N_*) (28)

Apparently, if the multiple degradation measures are independent, Equation (28) becomes:

(29) $$R(t)=P\left(X_{1}(t)<D_{1}\right)\times \cdots \times P\left(X_{N}(t)<D_{N}\right)=R_{1}(t)\times \cdots \times R_{N}(t)$$

where *R_p_*(*t*), *p* = 1, 2, …, *N* is the reliability function associated with the *p*th performance parameter under *S*_0_ as defined in Equation (16).

However, the multiple performance parameters are usually dependent. In order to address the dependency among multiple degradation processes, a copula function can be applied, and the best model can be selected based on AIC. Essentially, the system reliability at time *t* under *S*_0_ can be performed as [19,26,27,30]:

*R*(*t*) = *C*(*R*_1_(*t*), *R*_2_(*t*), …, *R_N_*(*t*); **δ**) (30)

where **δ** is the parameter set of a family of copulas.

### 3.3. Statistical Inference

Consider a copula-based multivariate distribution for random vector **X** with PDF:

(31) $$f\left(X;\Theta_{1},\ldots ,\Theta_{N},\delta \right)=c\left(F_{1}\left(X_{1};\Theta_{1}\right),\ldots ,F_{N}\left(X_{N};\Theta_{N}\right);\delta \right)\prod_{p=1}^{N}f_{p}\left(X_{p};\Theta_{p}\right)$$

where *F_p_*(; Θ*_p_*) and *f_p_*(; Θ*_p_*), *p* = 1,…,*N* are CDF and PDF of the *p*th marginal distribution with parameter set Θ*_p_* = {*θ_p_*, *γ_p_*, *σ_p_*, *a_p_*, *b_p_*}, *c*(;**δ**) is the density of copula function *C*(;**δ**) with parameter set **δ**.

For a sample of size *n*, the full log-likelihood function can be expressed as:

(32) $$\ln L\left(\Theta_{1},\ldots ,\Theta_{N},\delta \right)=\underset{Dependence\;structure\;L_{C}}{\underset{︸}{\sum_{i=1}^{n}\ln c\left(F_{1}\left(X_{1i};\Theta_{1}\right),\ldots ,F_{N}\left(X_{Ni};\Theta_{N}\right);\delta \right)}}+\underset{Marginals\;\sum_{p=1}^{N}L_{p}}{\underset{︸}{\sum_{p=1}^{N}\sum_{i=1}^{n}f_{p}\left(X_{pi};\Theta_{p}\right)}}$$

Clearly, the parameter set Ω = {Θ_1_, …, Θ*_N_*, **δ**} can be estimated by maximizing Equation (32). However, as the dimension of variables increases, it is difficult to obtain the optimal solution.

Joe [35] proposed a computationally attractive alternative to MLE, called Inference Functions for Margins (IFM) method, to estimate the parameters in multivariate copula models. The idea is to decompose Equation (32) into two parts: the contribution, denoted by *L_C_*, from the dependence structure in data represented by copula *C*, and the contributions, denoted by *L_p_*, *p* = 1, …, *N*, from each marginal distribution. Then, the approach estimates the parameters of marginal distributions and the copula in two stages. In the first stage, the parameters of each marginal distribution are estimated from the corresponding *L_p_*. In the second stage, the copula’s parameters are obtained by maximizing *L_C_* using the marginal distribution parameters estimated in the first stage.

In this paper, the IFM method is used to estimate the parameters Ω = {Θ_1_, …, Θ*_N_*, **δ**} of the copula-based multivariate CSADT model in the following two stages.

- *Stage 1. Parameter estimation of marginal distributions*.

For the CSADT data of each degradation measure, the parameter estimation method given in Section 2.3 can be used to estimate the parameter set Θ*_p_* = {*θ_p_*, *γ_p_*, *σ_p_*, *a_p_*, *b_p_*} of each marginal distribution by maximizing the log-likelihood function. Then, assuming that the failure threshold of the *p*th performance characteristic is *D_p_*, *p* = 1, 2, …, *N*, we can respectively get the reliability functions of all performance parameters under normal operating condition *S*_0_ using Equation (16).

- *Stage 2. Parameter estimation of copulas*.

Using the first-stage estimate ${\hat{\Theta }}_{p}$, the copula parameter set **δ** can be estimated by maximizing the copula likelihood contribution:

(33) $$\hat{\delta }=\arg\;\max\sum_{i=1}^{n}\ln c\left(R_{1}(X_{1i};{\hat{\Theta }}_{1}),\cdots ,R_{N}(X_{Ni};{\hat{\Theta }}_{N});\delta \right)$$

## 4. Case Study

In this section, a case study is used to demonstrate the validity and estimation performance of the proposed copula-based multivariate CSADT model and computational method.

### 4.1. Problem Description

**Figure 2:**
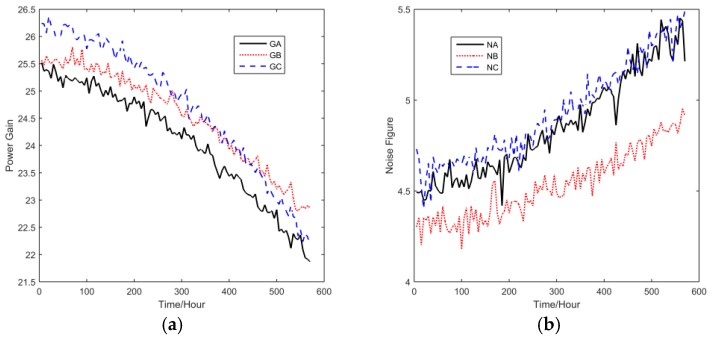
The performance parameters of a tuner. (**a**) Power gains; (**b**) Noise figures.

The tuner is a kind of microwave electronical assembly, which is required to have a long lifetime and high reliability. Previous failure analysis shows that temperature is the major factor that causes tuner output degradation. To quickly evaluate the lifetime and reliability of the tuner, a temperature CSADT with four stress levels was conducted. Six performance parameters of each tuner were measured by a computerized measuring system, including the power gain GA, GB, and GC, the noise figure NA, NB and NC, where G denotes power gain, N means noise figure, A, B and C indicate different directions. Figure 2 shows a sample from one tuner.

**Figure 3:**
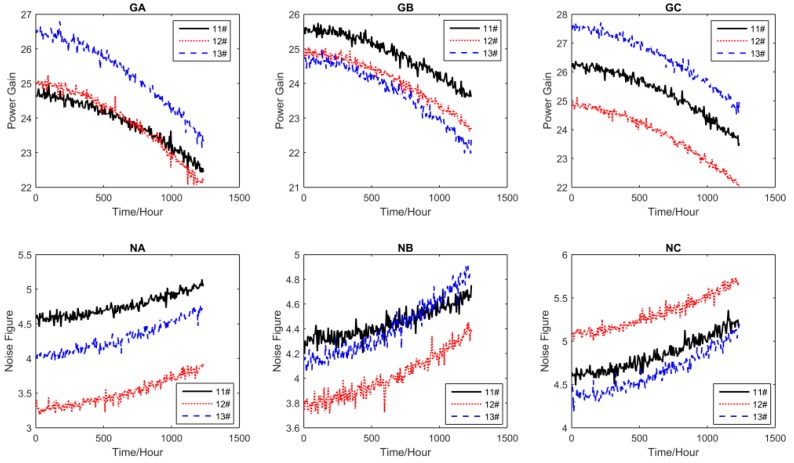
The accelerated degradation data of all parameters under S_1_.

**Figure 4:**
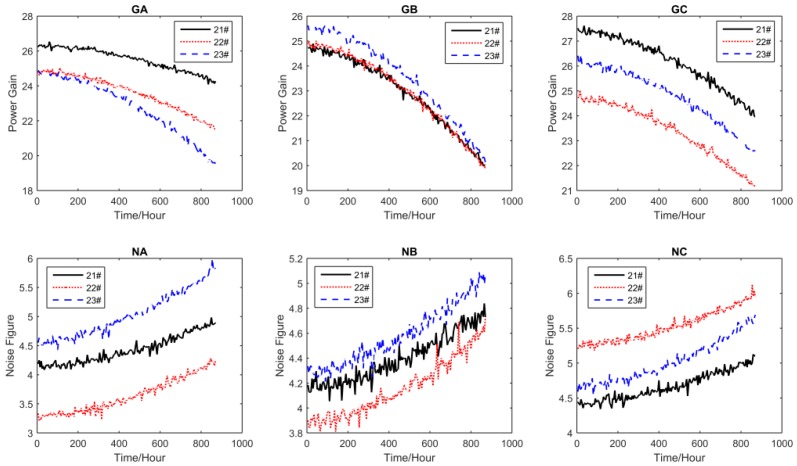
The accelerated degradation data of all parameters under S_2_.

**Figure 5:**
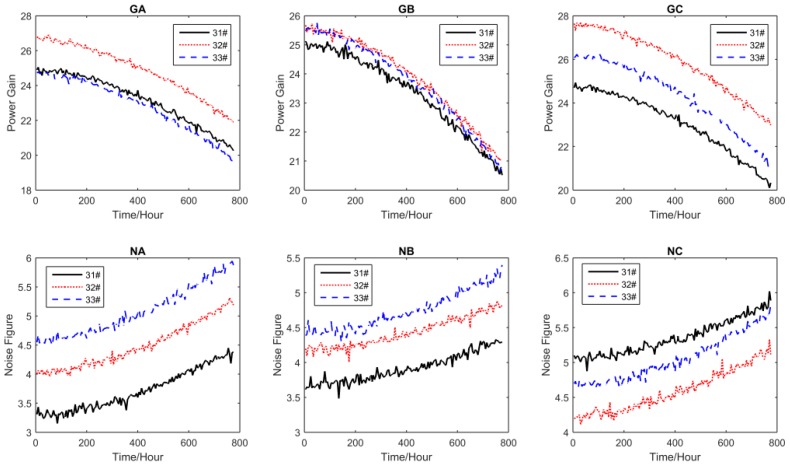
The accelerated degradation data of all parameters under S_3_.

**Figure 6:**
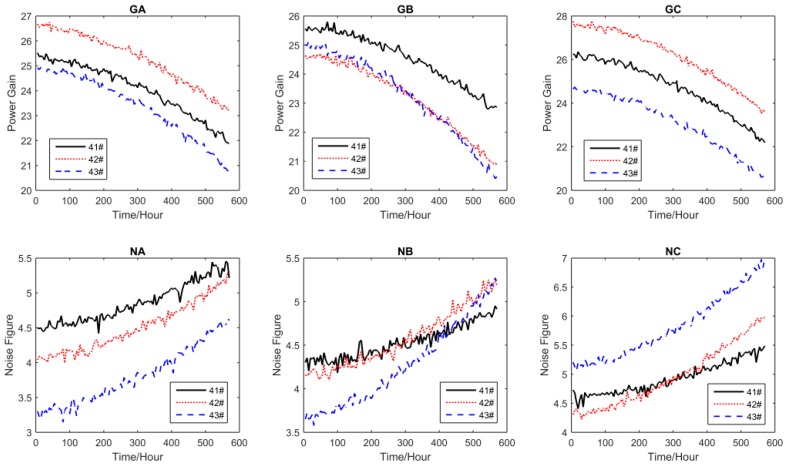
The accelerated degradation data of all parameters under S_4_.

For all performance parameters, the sampling interval was Δ*t* = 5 h. The other settings of the test are shown in Table 2, where S_1_, S_2_, S_3_, and S_4_ are the accelerated stress levels, and the normal operating condition is S_0_ = 25 °C. The accelerated degradation data of all performance parameters obtained by CSADT are shown in Figure 3, Figure 4, Figure 5 and Figure 6.

**Figure 7:**
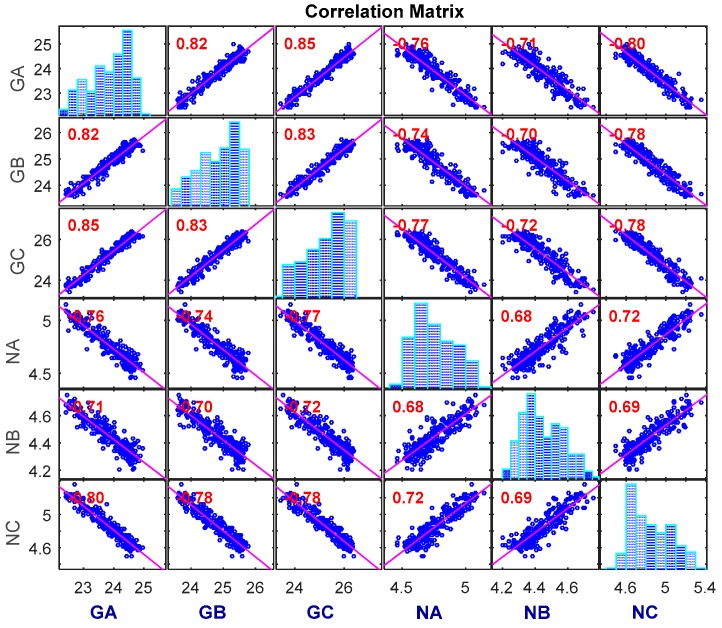
Kendall’s rank correlations between multiple degradation measures of a specimen.

Figure 7 gives a matrix of plots showing Kendall’s rank correlations among pairs of performance parameters of a specimen. The diagonal elements show the histograms of different degradation paths, and the off-diagonal elements are the scatter plots of pairs of performance parameters along with the Kendall’s rank correlation coefficients. From Figure 7, one can see that all pairs of degradation measures are significantly correlated. Therefore, in order to achieve more accurate and practical evaluation results of the tuner’s CSADT, it is worth considering the dependence among the multiple degradation processes.

### 4.2. Univariate ADT Models with General Wiener Process

First of all, the original degradation data of GA, GB, and GC are transformed by:

(34) $$y_{i}=\frac{x_{0}-x_{i}}{x_{0}}$$

where *x*_0_ and *x_i_* are the initial and *i*th degradation measurements.

Similarly, the original degradation data of NA, NB, and NC are also transformed by:

(35) $$y_{i}=\frac{x_{i}-x_{0}}{x_{0}}$$

Then, the proposed general Wiener process model, i.e., Equation (3), is used to fit the CSADT data of each performance parameter separately. For convenience and without loss of generality, the formulas Λ(*t*; *θ*) = *t^θ^* and *τ*(*t*; *γ*) = *t^γ^* are used in this paper according to the behavior of the degradation over time. It is important to mention that although we only focus on a combination of Λ(*t*; *θ*) = *t^θ^* and *τ*(*t*; *γ*) = *t^γ^*, other forms of Λ(*t*; *θ*) and *τ*(*t*; *γ*) can be adopted without extra theoretical difficulties, if applied [16]. The estimates of univariate ADT model parameters for each performance characteristic are obtained by the method provided in Section 2.3 and are listed in Table 3.

From Table 3, it can be seen that the estimate of *γ* for each performance parameter is close to 1, which indicates that the proposed model could be simplified. Simplify the univariate ADT model with the assumption of *γ* = 1, i.e., *τ*(*t*; *γ*) = *t*. The estimates of the simplified model (SM) parameters for each degradation measure are listed in Table 4.

To justify the use of the full model (FM) or the simplified model (assume *γ* = 1) for the univariate ADT data of each performance parameter, the likelihood ratio (LR) test [43] is implemented. The test statistic distribution has 1 degree of freedom, and the significance level is 5%. The result of the likelihood ratio test is shown in Table 5.

Since the critical value is $\chi_{1,0.05}^{2}=3.84$, we accept the assumption of *γ* = 1 except for GA. While, the *p*-value of GA is close to 0, which also indicates that there is strong evidence suggesting that the full model fits the GA data better than the simplified model. Hence, the proposed general Wiener process model with *τ*(*t*; *γ*) = *t^γ^* is used to fit the CSADT data of GA, and the simplified model (assume *γ* = 1) is used to fit the other performance parameters of tuner.

**Figure 8:**
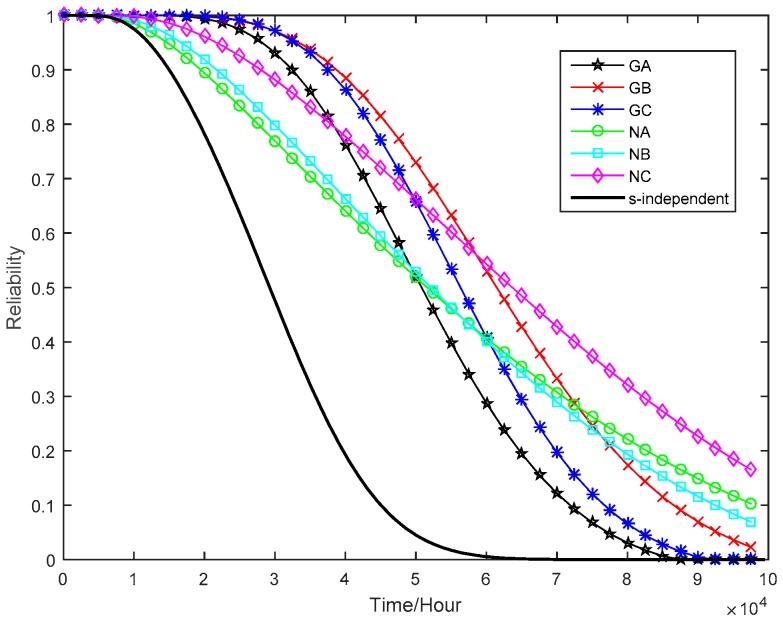
Marginal reliability function for each performance parameter.

For the failure thresholds of degradation measures, it is assumed that the tuner fails when one of the power gains, i.e., GA, GB, and GC, drops to 60% of its initial value. Meanwhile, the tuner would also fail if one of the noise figures, i.e., NA, NB, and NC, increases to 100% of its initial value. According to Equation (16), the marginal reliability functions of six performance parameters at the normal stress can be calculated, as shown in Figure 8. From Equation (29), we also estimate the system reliability of the tuner at the normal operating condition without considering the dependence among multiple performance parameters, which is displayed in Figure 8. From Figure 8, one can see that the tuner’s reliability is far less than the marginal reliabilities of individual degradation processes.

### 4.3. Multivariate Accelerated Degradation Model with Copulas

**Figure 9:**
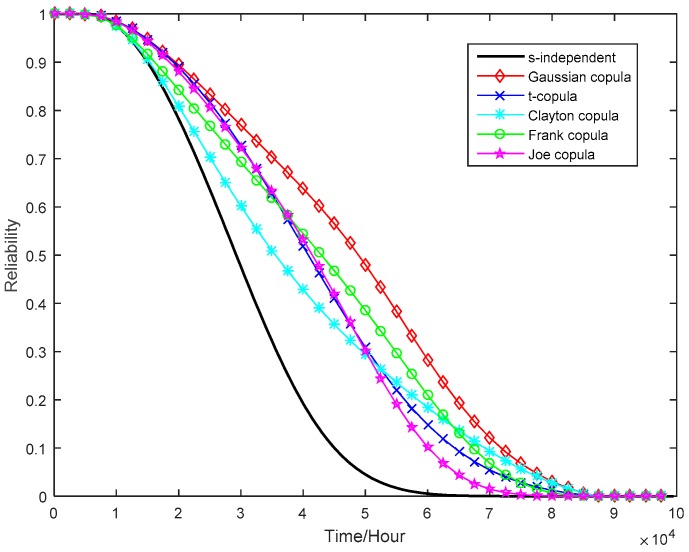
System reliability functions obtained using different multivariate copulas.

Based on the marginal reliability functions, the copula method is applied to fit the dependent multivariate ADT data. To select the best copula, the AIC is used when comparing candidate copulas. Five widely used multivariate copulas are selected to fit the joint distribution of multiple degradation processes. The results of parameter estimation and AIC values are summarized in Table 6, and the joint reliability curves of tuner with different copulas are shown in Figure 9.

As can be seen in Table 6, the multivariate ADT model with Frank copula has the smallest AIC. In other words, Frank copula may best describe the dependence among the multiple accelerated degradation processes. On the other hand, the ADT model with the Gaussian copula has the largest AIC, and thus is not adequate to describe the dependency between the two performance parameters by linear correlation [28].

**Figure 10:**
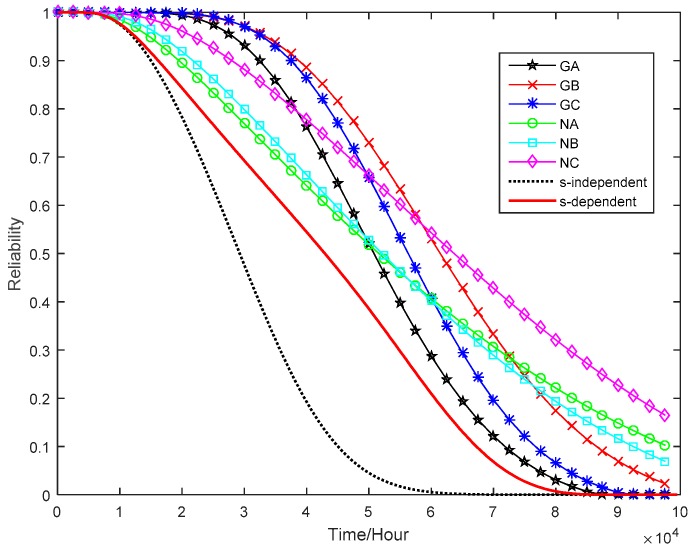
Comparison of reliability functions of tuner and its performance parameters.

Finally, the *s*-dependent system reliability of tuner is compared with that obtained under an *s*-independent assumption. From Figure 10, one can see that the *s*-dependent reliability curve is higher than s-independent reliability curve. In other words, making the *s*-independent assumption may be under-estimate the system reliability. This is not uncommon in many engineering applications. Therefore, it is more reasonable to use the proposed method in product reliability estimation when the strong dependency among multiple performance parameters exhibits.

## 5. Conclusions

In this paper, we have proposed a multivariate ADT model based on general Wiener process and copulas for a system with *s*-dependent nonlinear degradation processes. We first introduced a general Wiener process to describe univariate accelerated degradation process and provided the corresponding parameter estimation method. This model can cover the commonly used linear and time-scale transformed Wiener processes as its limiting cases. Then, the copulas were employed to develop a more flexible *s*-dependent multivariate ADT model, where the dependency among different degradation processes is linked by the copulas. The IFM method is applied to estimate the unknown parameters of copula and marginal distributions, and the AIC is used as the criterion to choose the best copula from several candidates. Finally, the case study validates the effectiveness and superiority of the general multivariate ADT model on solving real-world reliability engineering problems. It provides a useful tool for the reliability evaluation and the development of maintenance strategies for products with multiple performance parameters. This is also a new approach for information fusion in engineering practice. Our future research will be focused on the development of a model for analyzing multiple dependent nonlinear step-stress ADT data.

## Figures and Tables

**Table 1:** Summary of some multivariate copulas.

Copulas	*C*(*u*_1_,*u*_2_,…,*u_N_*)	Parameter
Gaussian Copula	$\Phi_{\rho }\left[\Phi^{-1}\left(u_{1}\right),\Phi^{-1}\left(u_{2}\right),\ldots ,\Phi^{-1}\left(u_{N}\right)\right]$ ^1^	ρ∈(−1,1)
Student’s *t*-Copula	$t_{\rho ,v}\left[t_{v}^{-1}\left(u_{1}\right),t_{v}^{-1}\left(u_{2}\right),\ldots ,t_{v}^{-1}\left(u_{N}\right)\right]$ ^2^	ρ∈(−1,1),ν>2
Clayton Copula	${\left(\sum_{i=1}^{N}u_{i}^{-\delta }-N+1\right)}^{-1/\delta }$	δ>0
Frank Copula	$-\delta^{-1}\log\left\{1+\frac{\Pi_{i=1}^{N}\left[\exp(-\delta u_{i})-1\right]}{{\left[\exp(-\delta )-1\right]}^{N-1}}\right\}$	$\begin{array}{l} \delta \in (-\infty ,\infty )\{0\}\\ \delta >0\;for\;N\geq 3 \end{array}$
Joe Copula	$1-{\left\{1-\Pi_{i=1}^{N}\left[1-{\left(1-u_{i}\right)}^{\delta }\right]\right\}}^{1/\delta }$	δ∈[1,∞)

^1^ Φ is the distribution function of a standard normal random variable, Φ*_ρ_* is the *N*-variate standard normal distribution with mean vector 0 and covariance matrix *ρ*; ^2^ *t_υ_* is a univariate *t* distribution with *υ* degrees of freedom, *t_ρ_*_,*υ*_ is the multivariate Student’s *t* distribution with a correlation matrix *ρ* with *υ* degrees of freedom.

**Table 2:** Testing parameters of tuners’ CSADT.

Stress Level	Temperature (°C)	Number of Monitoring	Sample Size
S_1_	55	247	3
S_2_	70	174	3
S_3_	80	155	3
S_4_	85	114	3

**Table 3:** Estimations of the univariate ADT model parameters.

Parameters	GA	GB	GC	NA	NB	NC
$\hat{\theta }$	1.6713	1.6648	1.6793	1.6136	1.6470	1.7285
$\hat{\gamma }$	1.0878	0.9815	1.0304	0.9730	0.9869	0.9861
$\hat{\sigma }$	0.1948	0.2835	0.2272	0.9380	0.8243	0.7258
$\hat{a}$	9.4654	11.6371	10.4689	11.5335	9.6690	12.7805
$\hat{b}$	−6234.05	−6956.20	−6599.09	−6633.39	−6152.89	−7400.12

**Table 4:** Estimations of the simplified univariate ADT model parameters (γ=1).

Parameters	GA	GB	GC	NA	NB	NC
$\hat{\theta }$	1.6810	1.6648	1.6805	1.6152	1.6455	1.7286
$\hat{\sigma }$	0.2625	0.2662	0.2520	0.8559	0.7886	0.6922
$\hat{a}$	9.4667	11.6342	10.4754	11.5638	9.6730	12.7771
$\hat{b}$	−6257.40	−6955.08	−6604.29	−6647.50	−6150.92	−7399.13

**Table 5:** Decision and *p*-value of the likelihood ratio test.

Contents	GA	GB	GC	NA	NB	NC
logL_FM	−1819.7	−1852.3	−1738.8	−4255.8	−4087.3	−3819.0
logL_SM	−1824.0	−1852.5	−1739.4	−4256.1	−4087.4	−3819.1
*p*-value	0.0034	0.5271	0.2733	0.4386	0.6547	0.6547
statstic	8.6	0.4	1.2	0.6	0.2	0.2
*h*	1	0	0	0	0	0

**Table 6:** Goodness-of-Fit for the copulas.

Copulas	Parameter Estimation	AIC	Ranking
Gaussian	*ρ*1 ^1^	21,783	5
Student’s *t*	[*ρ*2 ^2^, 98.106]	−5613	2
Clayton	2.15193	2910	4
Frank	9.3625	−13,141	1
Joe	3.165	1634	3

^1^ $\rho 1=\left[\begin{array}{cccccc} 1 & 0.97279 & 0.98800 & 0.96986 & 0.97379 & 0.96522\\ 0.97279 & 1 & 0.97609 & 0.99985 & 0.99997 & 0.99916\\ 0.98800 & 0.97609 & 1 & 0.97288 & 0.97668 & 0.96844\\ 0.96986 & 0.99985 & 0.97288 & 1 & 0.99977 & 0.99967\\ 0.97379 & 0.99997 & 0.97668 & 0.99977 & 1 & 0.99893\\ 0.96522 & 0.99916 & 0.96844 & 0.99967 & 0.99893 & 1 \end{array}\right]$; ^2^ $\rho 2=\left[\begin{array}{cccccc} 1 & 0.59714 & 0.88118 & 0.35281 & 0.39528 & 0.34503\\ 0.59714 & 1 & 0.73995 & 0.7129 & 0.74043 & 0.85853\\ 0.88118 & 0.73995 & 1 & 0.36259 & 0.40725 & 0.44296\\ 0.35281 & 0.7129 & 0.36259 & 1 & 0.99863 & 0.94762\\ 0.39528 & 0.74043 & 0.40725 & 0.99863 & 1 & 0.95163\\ 0.34503 & 0.85853 & 0.44296 & 0.94762 & 0.95163 & 1 \end{array}\right]$.

## Abbreviations

The following abbreviations are used in this manuscript:

ADT	Accelerated degradation testing
SLD	Super luminescent diode
MOSFET	Metal oxide semiconductor field effect transistor
IG	Inverse Gaussian
PDF	Probability density function
CSADT	Constant-stress accelerated degradation testing
SSADT	Step-stress accelerated degradation testing
FPT	First passage time
CDF	Cumulative distribution function
MLE	Maximum likelihood estimation
AIC	Akaike Information Criterion
IFM	Inference functions for margins
